# Safety and efficacy of direct Cardiac Shockwave Therapy in patients with ischemic cardiomyopathy undergoing coronary artery bypass grafting (the CAST-HF trial): study protocol for a randomized controlled trial

**DOI:** 10.1186/s13063-020-04369-0

**Published:** 2020-05-30

**Authors:** Leo Pölzl, Felix Nägele, Michael Graber, Jakob Hirsch, Daniela Lobenwein, Martina Mitrovic, Agnes Mayr, Markus Theurl, Michael Schreinlechner, Matthias Pamminger, Christian Dorfmüller, Michael Grimm, Can Gollmann-Tepeköylü, Johannes Holfeld

**Affiliations:** 1 University Clinic of Cardiac Surgery, Medical University of Innsbruck, Innsbruck, Austria; 2 Clinical Trial Center, Medical University of Innsbruck, Innrain 52, 6020 Innsbruck, Austria; 3 University Clinic of Radiology, Medical University of Innsbruck, Innsbruck, Austria; 4 University Clinic of Internal Medicine III, Medical University of Innsbruck, Innsbruck, Austria; 5 Heart Regeneration Technologies GmbH, Innsbruck, Austria

**Keywords:** Shockwave, CABG, Ischemic heart disease, Heart failure, Clinical trial

## Abstract

**Background:**

Coronary artery diseases (CAD) remains a severe socio-economic burden in the Western world. Coronary obstruction and subsequent myocardial ischemia result in progressive replacement of contractile myocardium with dysfunctional, fibrotic scar tissue. Post-infarctional remodeling is causal for the concomitant decline of left-ventricular function and the fatal syndrome of heart failure. Available neurohumoral treatment strategies aim at the improvement of symptoms. Despite extensive research, therapeutic options for myocardial regeneration, including (stem)-cell therapy, gene therapy, cellular reprogramming or tissue engineering, remain purely experimental. Thus, there is an urgent clinical need for novel treatment options for inducing myocardial regeneration and improving left-ventricular function in ischemic cardiomyopathy.

Shockwave Therapy (SWT) is a well-established regenerative tool that is effective for the treatment of chronic tendonitis, long-bone non-union and wound-healing disorders. In preclinical trials, SWT regenerated ischemic myocardium via the induction of angiogenesis and the reduction of fibrotic scar tissue, resulting in improved left-ventricular function.

**Methods/design:**

In this prospective, randomized controlled, single-blind, monocentric study, 80 patients with reduced left-ventricular ejection fraction (LVEF≤ 40%) are subjected to coronary-artery bypass-graft surgery (CABG) surgery and randomized in a 1:1 ratio to receive additional cardiac SWT (intervention group; 40 patients) or CABG surgery with sham treatment (control group; 40 patients). This study aims to evaluate (1) the safety and (2) the efficacy of cardiac SWT as adjunctive treatment during CABG surgery for the regeneration of ischemic myocardium. The primary endpoints of the study represent (1) major cardiac events and (2) changes in left-ventricular function 12 months after treatment. Secondary endpoints include 6-min Walk Test distance, improvement of symptoms and assessment of quality of life.

**Discussion:**

This study aims to investigate the safety and efficacy of cardiac SWT during CABG surgery for myocardial regeneration. The induction of angiogenesis, decrease of fibrotic scar tissue formation and, thus, improvement of left-ventricular function could lead to improved quality of life and prognosis for patients with ischemic heart failure. Thus, it could become the first clinically available treatment strategy for the regeneration of ischemic myocardium alleviating the socio-economic burden of heart failure.

**Trial registration:**

ClinicalTrials.gov, ID: NCT03859466. Registered on 1 March 2019.

## Background

### Myocardial Infarction and ischemic cardiomyopathy

Myocardial infarction (MI) caused by coronary artery disease (CAD) represents the leading cause of death in the European Union and the Western World [1, 2]. The prevalence is increasing due to a constantly aging population. Ischemia results in loss of cardiomyocytes and subsequent replacement of contractile myocardium with dysfunctional fibrotic scar tissue. Left-ventricular remodeling is causal for concomitant decline of left-ventricular function resulting in ischemic cardiomyopathy, the most common cause for heart failure [3]. Despite modern pharmacotherapy, patients with ischemic cardiomyopathy often exhibit a poor outcome and a markedly reduced quality of life. In addition, long hospital stays, rehabilitation, incapacity for work as well as the possible need for repeated intervention cause a severe socio-economic burden [4, 5].

Coronary-artery bypass-graft surgery (CABG) and percutaneous coronary intervention (PCI) represent the current state-of-the-art techniques for revascularization in patients with ischemic cardiomyopathy [6, 7]. Thereby, according to the data obtained from the STICH trial, patients with multivessel disease and reduced left-ventricular ejection fraction (LVEF) benefit from CABG surgery [8], as complete revascularization improves the long-term prognosis of patients with ischemic cardiomyopathy. However, although blood flow to ischemic myocardium is restored, revascularization represents a somewhat palliative approach, preventing repeated ischemic events, but hardly improving heart function [9–11]. Hence, new therapies, aiming to retrieve lost cardiac muscle are of considerable importance. Existing strategies, such as stem-cell or gene therapy, have shown promising results in pre-clinical and clinical trials. However, they fail to gain broad clinical use due to ethical concerns, complex preparation and application as well as a non-favorable side-effect profile [12]. Therefore, clinicians and patients alike are still in desperate need for a novel treatment option, capable of regenerating myocardium in patients with ischemic cardiomyopathy.

### Shockwave Therapy

Shockwaves represent a specific type of sound pressure waves that occur by a sudden release of energy, emerging, e.g., as thunder when lightning strikes. In medicine, shockwaves have been used for more than 30 years for disintegrating kidney stones (i.e., lithotripsy) [13]. The incidental finding of iliac-bone thickening after treatment led to the first applications of Shockwave Therapy (SWT) for bone regeneration in the early 1980s [14]. First results indicated improved healing of long-bone non-union after SWT. Thereby, SWT-induced bone-healing at only a tenth of the energy levels utilized in lithotripsy, allowing for an extension of indications for soft tissue pathology [15, 16]. Due to encouraging results, indications for SWT were extended to chronic tendonitis (e.g., tennis elbow), wound-healing disorders and erectile dysfunction [17]. Since then this novel approach of regenerative SWT finds use in a multitude of indications. Extensive basic research in the field showed release of angiogenic growth factors, recruitment of endogenous progenitor cells and modulation of the inflammatory response as the main underlying mechanisms responsible for the regenerative effects of SWT in the various pathologies.

Currently, Austria and Germany represent the leading countries in experimental and clinical shockwave research. Accordingly, reimbursement from health insurances has already been established for certain indications within German-speaking countries.

### Cardiac Shockwave Therapy

In 2005, the research group working with the coordinator of direct Cardiac Shockwave Therapy in patients undergoing coronary artery bypass grafting (CAST-HF) started to evaluate the potential effect of SWT for the regeneration of infarcted myocardium by direct epicardial application during open-heart surgery. Numerous pre-clinical experiments showed encouraging results in various animal models. Cardiac SWT was shown to increase the numbers of capillaries and arterioles in ischemic myocardium and, thus, reduced myocardial scar tissue. Moreover, the treatment prevented unfavorable left-ventricular remodeling and, thus, resulted in improved left-ventricular function in small- and large-animal models [18, 19]. As underlying causes, the group identified the release of angiogenic growth factors from the extracellular matrix, in particular vascular endothelial growth factor (VEGF), fibroblast growth factor (FGF) and placental growth factor (PlGF). Subsequent stimulation of VEGF receptor 2 (VEGFR2), inducing pro-angiogenic Akt/ERK signaling, caused new blood vessel formation by sprouting from existing vessels [20]. In addition to angiogenesis, SWT caused the release of stromal-cell-derived factor 1 (SDF-1), a crucial chemoattractant for the recruitment of endothelial progenitor cells. In a green fluorescent protein (GFP) bone-marrow-transplantation model we observed increased numbers of bone-marrow-derived endothelial cells at the site of ischemic injury clearly indicating postnatal vasculogenesis upon SWT [19]. Besides increased vascularization, we identified modulation of the inflammatory response as a crucial mechanism responsible for regenerative SWT effects. Modified cytokine release caused polarization of macrophages towards regenerative M2 macrophages, promoting regeneration [21].

Elucidating possible underlying mechanisms of the mechanotransduction responsible for the translation of the mechanical impulse into a biological response, we identified the release of angiogenic extracellular vesicles (EV) upon SWT. Released EVs activate Toll-like receptor 3 (TLR3), a receptor of the innate immune system recognizing danger-associated molecular patterns (DAMPS) released upon cellular stress [22]. Activation of TLR3 resulted in angiogenesis and regeneration of ischemic muscle, whereas SWT effects were almost completely abolished in *TLR3-*deficient animals [22]. However, the exact mechanics concerning the conversion of the physical stimulus to a cellular response (mechanotransduction) are not yet completely understood and will be a main target of the experimental research branch inherent in CAST-HF.

### Pilot trial

The aforementioned preclinical findings provided the basis for translation into a clinical setting. In a first-in-man pilot study in 2008, ten patients with severe left-ventricular dysfunction due to a post-infarctional transmural scar and an indication for CABG received direct epicardial SWT in addition to standard CABG surgery. Direct cardiac shock-wave therapy was successfully performed in all patients. There were no severe side-effects observed neither upon treatment (intraoperative arrhythmias, cardiac hematoma formation, lacerations with causal relation to shock-wave therapy), nor in the 6-month follow-up period (data not published).

Hence, this first-in-men application proved safety and feasibility of the new therapy and the underlying medical device. We therefore think that direct cardiac SWT may develop as a safe and cost-effective adjunctive therapy to CABG, in particular for patients with large areas of dysfunctional myocardium due to ischemia. To date, no published data exist on direct cardiac SWT in humans.

### The CAST-HF trial

The current trial (CAST-HF) is a monocenter, prospective, randomized controlled, single-blinded study.

In this study, the following null-hypothesis will be tested: There will be no difference in LVEF between the two study groups 1 year after treatment.

Alternative hypothesis: There will be a significant increase of the primary endpoint, LVEF in the shockwave group compared to the control group 1 year after treatment.

### Study design

**Fig. 1:**
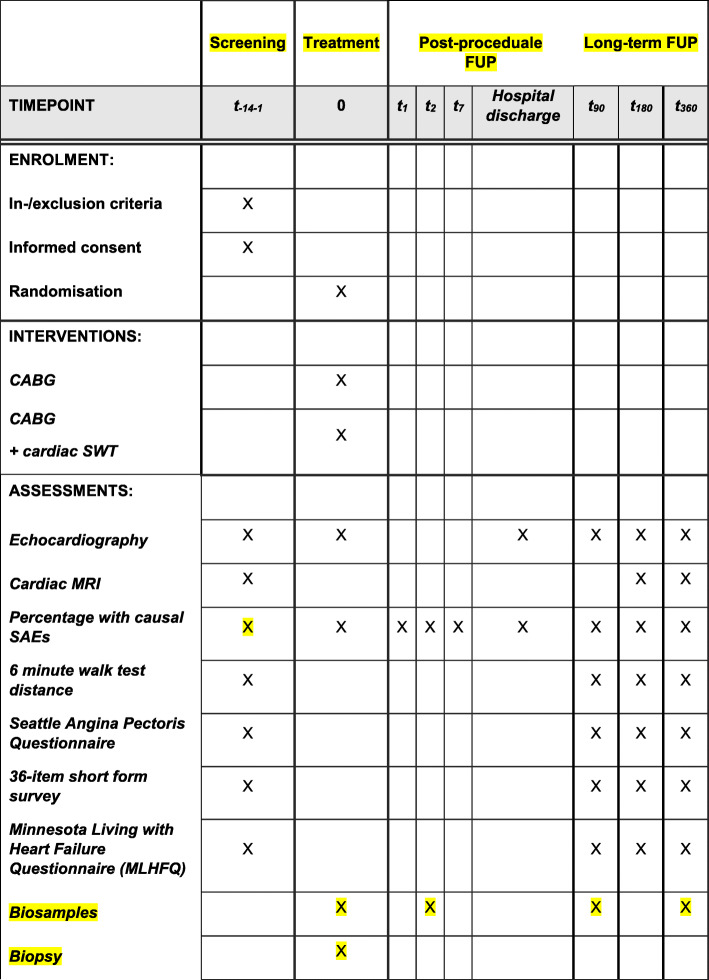
Standard Protocol Items: Recommendations for Interventional Trials (SPIRIT) Figure

Safety and efficacy of direct Cardiac Shockwave Therapy in patients undergoing coronary artery bypass grafting (CAST-HF) is a prospective, single-blind, randomized controlled, single-center study assessing the efficacy and safety of Cardiac Shockwave Therapy adjunctive to CABG. Eighty male or female patients aged above 21 years and under 80 years of age with reduced left-ventricular function (LVEF ≤ 40%) and regional left-ventricular-wall-motion abnormalities undergoing primary CABG are randomly assigned in a 1:1 ratio to receive additional cardiac SWT (intervention group; 40 patients) or sham treatment (control group; 40 patients). For the sham treatment, a non-functional applicator will be held on the exact same areas of the heart for the same time as in the treatment group (Fig. 1).

Screening of patients for trial inclusion will be performed by physicians. For each case, after having been indicated by the cardiac team the responsible cardiac surgeon will set the final indication for CABG. LVEF will be assessed by cardiac magnetic resonance imaging (MRI). Detailed study inclusion and exclusion criteria are listed in the Table 1 below.

**Table 1:** Patient inclusion and exclusion criteria for the CAST-HF trial

Inclusion criteria	Exclusion criteria
• Male or female patients above 21 years and under 90 years of age undergoing primary coronary artery bypass grafting • Patients must present with reduced left-ventricular function defined as LVEF ≤ 40% • Patients must present with regional left-ventricular-wall-motion abnormalities • Patients have to give written informed consent to participate in the study	• Significant concomitant aortic valve disease in need of surgical treatment (except significant aortic valve disease not detected in preoperative cardiac ultrasound that is detected intra-operatively) • Serious radiographic contrast allergy • Patients in cardiogenic shock • Patients with contraindication for cardiac MRI • History of significant ventricular arrhythmias, except arrhythmias associated with MI • Present co-morbidity which reduces life expectancy to less than 1 year • Presence of a ventricular thrombus • Presence of a cardiac tumor • Pregnancy

Key: *LVEF* left ventricular ejection fraction, *MI* myocardial infarction, *MRI* magnetic resonance imaging

Physicians will inform patients about the possibility of participation in the study. On the Consent Form, participants will be asked:

- If they agree to use of their data should they decide to withdraw from the trial
- If they agree that their data and blood as well as myocardial samples can be used for future cardiac research projects

(The model Informed Consent Form is available from the corresponding author on request.)

The primary aim of the study is to investigate the difference in the primary endpoint, LVEF between the control and the shockwave group 1 year after treatment. Secondary aims include safety, global and regional viability, assessment of heart failure symptoms as well as quality of life according to the study protocol. Detailed study endpoints are listed below.

### Primary endpoint

1. Improvement of LVEF (Day (D)7)
2. The percentage of participants experiencing a major adverse event (D90)

### Secondary endpoints

1. Endpoints related to the safety during the treatment phase are as follows: The percentage of participants experiencing a major adverse events (time frame: up to D360)
2. Endpoints related to the efficacy as follows: Improvement of LVEF (D90, D180, D360); Improvement in the 6-minute Walk Test distance (D7, D90, D180, D360); Improvement of the patient’s physical limitations caused by angina, the frequency of and recent changes in their symptoms, their satisfaction with treatment, and the degree to which they perceive their disease to affect their quality of life, as assessed with the Seattle Angina Pectoris Questionnaire (D7, D90, D180, D360); Improvement in quality of life as assessed by the 36-item short form health survey (SF36) (D7, D90, D180, D360); and Improvement in quality of life as assessed by the Minnesota Living with Heart Failure Questionnaire (MLHFQ) (D7, D90, D180, D360)

### Biosampling

To identify a biomarker for the efficacy of SWT, blood and urine samples of all patients will be collected on D-1; D0, D3, D90 and on D360. Cells of samples, whole blood, serum, plasma, DNA and RNA, will be stored in a biostorage system acquired for this study. In summary there will be 14,400 samples collected and stored during the trial. In addition, 3 epicardial biopsies will be taken before SW or sham treatment and 3 epicardial biopsies 15 minutes thereafter.

During this study an extensive heart failure/CABG Bio-Bank will be archived and consequently offers the possibility to study unrevealed aspects of surgical heart failure therapy with and without SWT. More details on sample handling and procedures are available from the corresponding author on request.

Duration of the planned recruitment phase consists of 24 months. The study will end 12 months after inclusion of the last patient. Evaluation times are separated in three periods: Screening and baseline period (day − 14 to day 1), treatment period (either CABG + direct epicardial shockwave therapy (DESWT) or CABG + sham treatment) including post-procedural follow-up (day 1 to hospital discharge) and long-term follow-up period (day of discharge to day 360). Time points of follow-up visits were chosen closely to the usual pathway to improve patient compliance. Patients who withdraw their consent, will be asked to attend for one unscheduled, final visit.

### Description of the device and its intended purpose

**Fig. 2:**
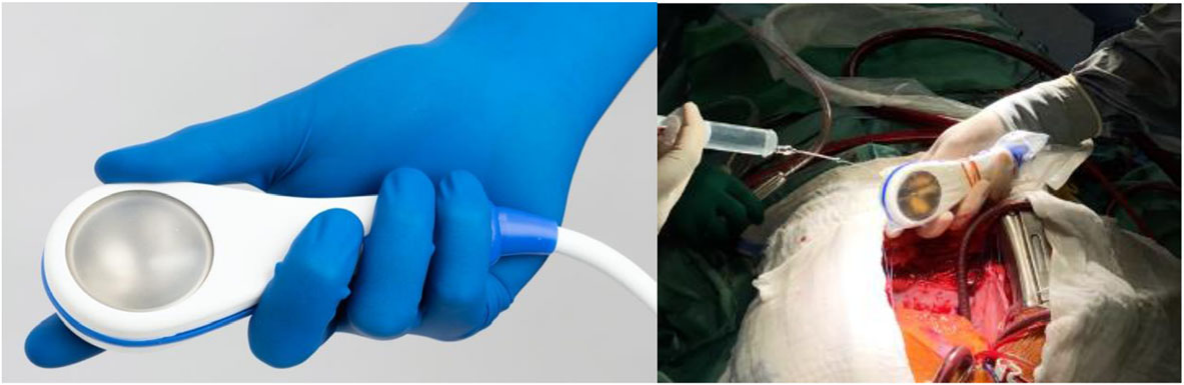
Cardiac shockwave applicator

Shockwaves will be generated and applied using a Cardiac Shockwave Probe (CSP) (Heart Regeneration Technologies GmbH, Innsbruck, Austria). This direct Cardiac Shockwave Therapy system is intended for shockwave application for therapeutic purposes of tissue regeneration of the myocardium. The commercially available device Flashwave MMC is a portable unit consisting of a basic housing unit, an electro-hydraulic shockwave generator, a control unit and a control panel (Nonvasiv Medical GmbH, Konstanz, Germany). The hand-held applicator CSP, which is connected to the Flashwave MMC via a removable cable, delivers the shockwaves to the patient via a water-filled coupling membrane and is provided as non-sterile for single use only (Fig. 2).

Prior to the application, the CSP is inserted into a sterile removable cover intended for intraoperative use (Civco Medical Solutions, 2301 Jones Blvd., Coralville, IA 52241, USA). The Flashwave MMC has several energy levels for the generation of shockwaves, and a varied frequency setting of 1 to 8 Hz (one to eight shockwave releases per second). For the application during the clinical study the energy level is fixed at the highest setting of the device in order to provide a shockwave energy flux density of 0.36 mJ/mm^2^, the level which has been used in the prior safety and feasibility study. The shockwave frequency is set to 4 Hz. CSP has direct contact with the epicardium via the sterile cover using sterile ultrasound gel in the cover.

Shockwave Therapy is performed after CABG surgery, while the patient is still on cardiopulmonary bypass. Three hundred shots are applied directly to each defined region of the ischemic myocardium. The application will take approximately 10–15 min.

The implementation of Cardiac Shockwave Therapy will not require alteration to usual care pathways, and these will continue for both trial arms.

### Data safety monitoring board

In order to ensure completely independent decision-making about the inclusion or exclusion of patients, as well as early study termination, an external Data Safety Monitoring Board (DSMB) has been established including independent experts who will review a patient’s data prior to decision-making about inclusion or exclusion in the study. The decision of the DSMB is documented on a DSMB decision form and is considered not to be a recommendation but mandatory for the investigators. This will overcome all conflict of interest issues by the investigators of the study.

### Randomization

This is a prospective, randomized controlled, single-blind, monocentricr study. The investigated medicinal treatment (SWT) to which the individual patients will be assigned is determined in a randomized fashion with a 1:1 allocation ratio. The randomization process has been realized independently from the clinical investigators. An independent statistician from an external company has created a randomization list. Surgeons receive allocation information (via envelopes) but not till the patient has already undergone CABG surgery in order to avoid bias related to the surgical procedure. Patients can only be unblinded after they have completed the follow-up visits or withdraw their participation. There will be no special criteria for discontinuing or modifying the allocated interventions.

### Statistical analysis

#### Power calculation

Assuming an effect size (Cohen’s d) of 0.678 for baseline/follow-up differences between groups on LVEF, 72 participants (36 intervention/36 control) are needed for statistical analysis with an adequate power, assuming an alpha risk of 0.05 (two-sided) and a beta risk of 0.20 (non-normal distributed). With an estimated loss to follow-up (mortality and dropout) of 10%, 80 patients have to be enrolled to ensure that 72 patients can be analyzed. Descriptive statistics will be performed for demographics and participant characteristics.

### Data analysis

#### Demographic and baseline characteristics

Demographic characteristics as well as administered study treatment, medical history, disease duration, use of prior treatments and concomitant medications will be summarized for all patients enrolled using appropriate descriptive statistics, i.e., number (%) of patients for categorical variables and mean, SD, median, minimum/maximum for continuous variables. Chi-squared tests and an independent *t* test will be used to determine between-group differences in baseline characteristics.

### Efficacy analysis

The primary efficacy variable is the LVEF absolute change from baseline, i.e., the intra-patient difference between the pre-treatment value (T1) and the value measured at the last examination (T2). This variable is quantitative and continuous and will be presented as mean value with 95% confidence intervals. A two-sided independent *t* test will be applied to test the following hypothesis:

$$ \kern0.5em H0:\mu\ \varDelta LVEFcontrol=\mu\ \varDelta LVEFshockwave $$

$$ \kern0.5em H1:\mu\ \varDelta LVEFcontrol\ne \mu\ \varDelta LVEFshockwave. $$

In case of non-normal distribution of LVEF a non-parametric test will be applied. Additionally a two-way repeated measures analysis of variance (ANOVA) (group x time) will be performed. For paired *t* tests and a repeated measures ANOVA, effect sizes will be calculated.

The same variable is documented at all study endpoints (6 months, 12 months after SWT). Although numerous interventions after admission may impact on the myocardial contraction, it may be important to see, for each individual patient, the evolution of LVEF over time. This may be a graphic representation, with the major interventions superposed. As applicable, a two-way ANOVA for repeated measurements or a linear mixed-effects model will be applied in order to analyze repeated measurements of LVEF. Alpha-corrected paired *t* tests (Bonferroni corrected) will be executed for localizing temporal differences shown by repeated measures ANOVAs.

### Safety and secondary analysis

All secondary and safety parameters will be summarized using descriptive statistics, i.e., number (%) of patients for categorical variables and mean, SD (standard deviation), median, minimum/maximum for continuous variables. Descriptive statistics will be produced by treatment group. No formal hypothesis testing will be performed. Appropriate statistical tests will be applied in an explorative manner only.

### Sex-specific analysis

Descriptive statistics including the estimate of variance or SD (as applicable) by sex will be reported. At the primary follow-up time point, regardless of the potentially limited statistical power of these sex-specific subgroup analyses, data will be examined for clinically meaningful sex differences in each of the following:

- Primary effectiveness endpoint
- Primary safety endpoints; and
- Key secondary endpoints

After overall effectiveness and safety have been investigated, the influence of sex on primary endpoints for both safety and effectiveness will be assessed.

### Interim analysis

Due to ethical considerations (implementing no more participants than necessary) an interim analysis will be performed after 40 participants in order to stop the recruiting process when sufficient statistical power has been reached. In the course of this analysis effect sizes found for the study participants will be compared with those from the a-priori sample size calculation. If there are strong discrepancies between the effect sizes the study should be treated as follows:

1. If the effect sizes from the interim analysis (ES_interim) is similar to those from the sample size calculation (*ES_samplesize*): the study should be contained till the end (80 participants)

$$ \mathrm{Requirement}: ES\_ samplesize\ast 0.85> ES\_ interim<1.25\ast ES\_ samplesize $$

2) If the effect size from the interim analysis is appreciably higher than those from the sample size calculation: a sample size calculation based on the novel effect (safety cushion 10%; effect size calculation new = 0.9 * *ES_interim*) should be analyzed. The result of this sample size calculation defines the maximum study cohort size.

$$ \kern1em \mathrm{Requirement}: ES\_ interim>1.25\ast ES\_ samplesize $$

3) If the effect size from the interim analysis is appreciably smaller than that from the sample size calculation: a second-step analysis as follows should be executed:

$$ \kern0.em \mathrm{Requirement}: ES\_ interim<0.75\ast ES\_ samplesize $$

- If there are significant deteriorations in the primary outcomes: closing the study, as the intervention induced a deterioration compared to the traditional treatment
- If there are significant improvements on the primary outcomes which can be proven by a sufficient statistical power (> 0.80), but the baseline/follow-up differences between groups leading to small and medium effects (Cohen’s d < 0.8): closing the study due to ethical reasons, as the maximum study size (*n* = 80) will not have the possibility to reach sufficient statistical power
- If there are significant improvements on the primary outcomes which cannot be proven by a sufficient statistical power (< 0.80): the study should be contained till the end (80 participants)

### Clinical trial conduct and data management

The procedures set out in this study protocol are designed to ensure that the sponsor and the investigator abide by the principles of the EN ISO 14155 recommended by the European Committee for Standardization and the Declaration of Helsinki concerning the conduct, evaluation and documentation of the study. The study will also be performed adhering the local legal conditions and requirements. Each investigator had to confirm this by signing the study protocol. Prior to study start, the study protocol and/or other appropriate documents were approved by the appropriate ethics committee and competent authorities. In case of any harm from participation in the trial, participants are insured for the length of their participation and 3 years after. No further payments or provisions are planned. The study is performed monocentrically at the University Clinic of Cardiac Surgery Innsbruck, Austria.

### Monitoring

Legally required monitoring visits will take place four times a year and according to the risk-based approach, whenever needed in between. Monitoring will be performed by the Clinical Trial Center of Medical University Innsbruck. Additional meetings, trainings, lists and standard operating procedures (SOP) help to increase the adherence to the protocol. According to the Austrian Medical Device Act the principle investigator is liable to cooperate with competent authority regarding inspections.

### Handling of data

Data will be collected in paper Case Report Forms and handled according to General Data Protection Regulation (GDPR) implemented on 25 May 2018. Data checks are performed constantly by the monitoring team. Plausibility checks will be performed before the analyses.

After closure of the CAST-HF, trial documents will be archived according to Austrian Law.

### Handling of missing data

A last-observation-carried-forward (LOCF) procedure will be applied in case of withdrawals.

## Trial status

The trial protocol has been approved by Ethics Committee of Medical University Innsbruck (reference number: EK 1118/2018) and by the Austrian Federal Office for Safety in Health Care (reference number: BASG 11262210). The study initiation was held in November 2018, the first patient was recruited in December 2018 and patient recruitment is scheduled to be finished within December 2020. Current study protocol version number 1.3 (effective date: 1st Dec 2019) is basis for this manuscript and written in accordance with the Standard Protocol Items: Recommendations for Interventional Trials (SPIRIT) Statement.

### Protocol amendments

At the end of 2019, we decided to implement:

1. Sham treatment for patients in the control arm
2. The inclusion age maximum was changed from 80 to 90 years
3. The addition of a left-ventricular biopsy
4. Intraoperative randomization
5. Change of study short title from CAST to CAST-HF

Amendments were informed to the Ethics Committee and to the competent authority (approved on 15 April 2020).

## Discussion

Ischemic cardiomyopathy due to coronary artery disease remains a major burden for affected patients and, thus, represent a major challenge for Western health care systems. Post-infarctional remodeling and replacement of contractile myocardium with dysfunctional scar tissue leads to alteration of left-ventricular geometry and, hence, cardiac output for organ perfusion resulting in heart failure. Congestion, fatigue, dyspnea and angina severely impact on patients’ quality of life and can cause repeated episodes of cardiac decompensation concomitant with the need for hospital admission. Patients with heart failure due to ischemic cardiomyopathy have a decreased life-expectancy [3].

Current neurohumoral treatment strategies mainly aim at the improvement of symptoms. Complete revascularization of viable myocardium remains a cornerstone in the treatment of ischemic cardiomyopathy. The available scientific evidence currently favors CABG over PCI in patients with multivessel disease and impaired LV systolic function [23]. However, CABG surgery represents a somewhat palliative strategy, as it mainly aims at avoiding novel MI rather than regenerating myocardium and improving contractility.

SWT represents a promising therapeutic tool for the regeneration of dysfunctional tissue. It has proven effective in numerous pathologies, mainly by induction of neovascularization and modulation of inflammation. Extensive preclinical studies in small animals as well as large animals show a clear benefit of SWT for the functional restoration of ischemic myocardium. For this purpose, the CAST-HF trial was initiated in 2018, representing the first randomized controlled trial to evaluate the benefit of direct cardiac shockwave therapy. Based on well-described evidence from in-vitro as well as numerous small and large animal experiments, this trial marks the next milestone to develop direct cardiac SWT for broad clinical routine use in patients suffering from ischemic heart failure [15–18, 21, 24, 25]. Thus, it could become the first available treatment option for the regeneration of ischemic myocardium. In contrast to other experimental treatment strategies, SWT has been used for many decades in medicine and to date there are no unfavorable long-term side-effects reported. In contrast, other promising experimental approaches for myocardial regeneration could not be translated into a clinical setting. Stem cells for myocardial regeneration have been investigated intensively. Despite promising preclinical results, the clinical translation has not been successful so far due to reports of arrhythmogenic events, lack of efficacy, failed incorporation into the site of injury or having malignant potential. In addition, severe allegations of scientific misconduct have caused the cancellation of large-scale trials further nourishing the doubts over the efficacy of stem-cell therapy for cardiac regeneration [26]. Another approach for myocardial regeneration remains the reprogramming of fibroblasts within the dysfunctional scar tissue towards functional cardiomyocytes using the Yamanaka factors. Again, apart from promising results in in-vitro and first-animal trials, the clinical translation remains far from close, as the method of delivery of the Yamanaka factors and the reprogramming efficacy remain questionable [27, 28].

Successful application of transthoracic SWT for the treatment of angina and left-ventricular dysfunction has been described. However, in contrast to direct epicardial SWT, the application window of transthoracic SWT remains limited due to the adjacent lung tissue. Due to the physical properties of shockwaves, they are absorbed by air simultaneously to ultrasound waves. In contrast, direct epicardial application enables treatment of the entire myocardium. In addition, direct coupling of the SWT applicator with the epicardium might ensure the more efficient delivery of SWT impulses [29, 30].

Although our group could elucidate the fundamental effects underlying the regenerative potential of SWT and found clear hints for the translation of the mechanical impulse to a biological response the exact underlying mechanism remains to be uncovered. The release of microvesicles (exosomes) containing angiogenic cargo and the subsequent stimulation of immune receptor Toll-like receptor 3 have been found. The origin of exosomes as well as the exact pathway from TLR3 induction to tissue regeneration will still be a major experimental research effort in parallel to CAST-HF. Exact knowledge of the underlying mechanism is of pivotal importance to establish further SWT options. This might possibly allow SWT not only during CABG, but also as an non-invasive standalone therapy via a minimally invasive cardiac applicator.

In conclusion, CAST-HF represents the first trial investigating the efficacy of direct cardiac SWT for the improvement of left-ventricular function in patients with ischemic cardiomyopathy. If positive, this trial could introduce SWT for broad clinical application, thus representing the first available therapeutic strategy for the regeneration of ischemic myocardium. Millions of affected patients could benefit from SWT effects and overcome ischemic heart failure. In addition, further knowledge of cardiac SWT could help develop new approaches and indications for SWT.

## Data Availability

The datasets analyzed during the current study are available from the corresponding author on reasonable request.

## Abbreviations

CABG: Coronary-artery bypass-graft surgery
CAD: Coronary arterial disease
CAST-HF: Direct Cardiac Shockwave Therapy in patients undergoing coronary artery bypass grafting
CSP: Cardiac Shockwave Probe
DAMPS: Danger-associated molecular patterns
DESWT: Direct epicardial shockwave therapy
DSMB: Data Safety Monitoring Board
EF: Ejection fraction
EV: Extracellular vesicles
FGF: Fibroblast growth factor
LVEF: Left-ventricular ejection fraction
MI: Myocardial infarction
MLHFQ: Minnesota Living with Heart Failure Questionnaire
MRI: Magnet resonance imaging
PCI: Percutaneous coronary intervention
PlGF: Placental growth factor
SDF-1: Stromal-cell-derived factor 1
SWT: Shockwave Therapy
TLR3: Toll-like receptor 3
VEGF: Vascular endothelial growth factor
VEGFR2: VEGF receptor 2
